# Sex Chromosomes of the Iconic Moth *Abraxas grossulariata* (Lepidoptera, Geometridae) and Its Congener *A. sylvata*

**DOI:** 10.3390/genes9060279

**Published:** 2018-05-31

**Authors:** Magda Zrzavá, Irena Hladová, Martina Dalíková, Jindra Šíchová, Erki Õunap, Svatava Kubíčková, František Marec

**Affiliations:** 1 Faculty of Science, University of South Bohemia, Branišovská 1760, 37005 České Budějovice, Czech Republic; magda.zrzava@gmail.com (M.Z.); irena.hladova@entu.cas.cz (I.H.); m.dalikova@gmail.com (M.D.); 2 Biology Centre of the Czech Academy of Sciences, Institute of Entomology, Branišovská 31, 37005 České Budějovice, Czech Republic; sichjindra@seznam.cz; 3 Institute of Ecology and Earth Sciences, University of Tartu, Vanemuise 46, 51014 Tartu, Estonia; erki.ounap@ut.ee; 4 Institute of Agricultural and Environmental Sciences, Estonian University of Life Sciences, Fr. R. Kreutzwaldi 5, 51014 Tartu, Estonia; 5 Veterinary Research Institute, Hudcova 70, 62100 Brno, Czech Republic; kubickova@vri.cz

**Keywords:** *Abraxas*, chromosome painting, comparative genomic hybridization, female heterogamety, heterochromatin, molecular divergence dating, ribosomal DNA (rDNA)

## Abstract

The magpie moth, *Abraxas grossulariata*, is an iconic species in which female heterogamety was discovered at the beginning of the 20th century. However, the sex chromosomes of this species have not yet been cytologically identified. We describe the sex chromosomes of *A. grossulariata* and its congener, *A. sylvata*. Although these species split only around 9.5 million years ago, and both species have the expected WZ/ZZ chromosomal system of sex determination and their sex chromosomes share the major ribosomal DNA (rDNA) representing the nucleolar organizer region (NOR), we found major differences between their karyotypes, including between their sex chromosomes. The species differ in chromosome number, which is 2*n* = 56 in *A. grossularita* and 2*n* = 58 in *A. sylvata.* In addition, *A. grossularita* autosomes exhibit massive autosomal blocks of heterochromatin, which is a very rare phenomenon in Lepidoptera, whereas the autosomes of *A. sylvata* are completely devoid of distinct heterochromatin. Their W chromosomes differ greatly. Although they are largely composed of female-specific DNA sequences, as shown by comparative genomic hybridization, cross-species W-chromosome painting revealed considerable sequence differences between them. The results suggest a relatively rapid molecular divergence of *Abraxas* W chromosomes by the independent spreading of female-specific repetitive sequences.

## 1. Introduction

In eukaryotes, two distinct modes of chromosomal sex determination have evolved, male heterogamety with XX/XY (female/male) and female heterogamety with WZ/ZZ (female/male) sex chromosome systems. Derived systems have evolved within each mode, including those lacking the Y or W sex chromosome or those with neo-sex chromosomes and multiple sex chromosomes. Male heterogamety was first described based on cytological observations by Stevens in 1905 [1], who found that males and females of the mealworm beetle, *Tenebrio molitor*, have the same number of chromosomes but differ by one pair of heteromorphic chromosomes that segregate to the opposite poles in meiotic anaphase I. Male heterogamety was later confirmed in *Drosophila melanogaster* by studies of sex-linked inheritance and named the XY system [2]. Female heterogamety was proposed at almost the same time by Leonard Doncaster in the magpie moth, *Abraxas grossulariata*. In 1906, Doncaster and Raynor published a paper on the inheritance of a pale variety of the magpie moth (lacticolor) that occurs more frequently in females than in males [3]. Later, inspired by Spillman’s theory of the female-limited ‘X’ chromosome, Doncaster proposed lacticolor as a sex-linked trait and suggested that its higher frequency in females is caused by female heterogamety in this species [4]. Despite a thorough cytogenetic investigation, he failed to support this theory with cytogenetic observations; both sexes had the same chromosome numbers, with no conspicuous heteromorphism. However, the presence of heterochromosomes was soon demonstrated in females of another moth, the ruby tiger moth, *Phragmatobia fuliginosa* [5]. The W and Z symbols used in the fundamental work of Morgan and colleagues [2] were then firmly established for sex chromosomes in gonochoristic organisms with female heterogamety.

During the 112 years since Doncaster’s and Raynor’s work, extensive research on sex chromosomes in moths and butterflies (Lepidoptera) has revealed that heterogametic females are indeed common, and probably universal, in this insect order [6,7,8], and their importance for adaptation, speciation, and sex determination has been repeatedly demonstrated in various species [9,10,11,12,13]. Female heterogamety also occurs in other groups of organisms, especially in non-mammalian vertebrates, including birds and snakes, and some lizards, turtles, amphibians, and fish [14,15]. In invertebrates, however, it has evolved only in a few phylogenetically distant groups. In insects, female heterogamety is characteristic of caddisflies (Trichoptera), a sister group of Lepidoptera [16]. As an exception, in the insect order Diptera, female heterogamety was reported for eight species of fruit flies of the family Tephritidae [17,18], but has not yet been confirmed in a detailed study. Female heterogamety has been demonstrated in some species of woodlice (Crustacea: Isopoda) [19], parasitic fluke worms of the family Schistosomatidae, Trematoda [20], and some snails, Gastropoda [21]. Accumulating evidence suggests its occurrence in another crustacean group, macruran Decapoda such as crayfish, shrimp, and prawns [22,23]. Finally, female heterogamety has evolved several times independently in a few plant species [24], though it appears to be less common than XY systems. 

The order Lepidoptera, with about 160,000 described species [25], is by far the largest animal taxon with female heterogamety [6]. Although only a small fraction of lepidopteran species has been examined, the available data suggest that most moths and butterflies have a WZ/ZZ system, except for basal lineages that share the absence of the W chromosome with Trichoptera [6,8]. Other exceptions include species with multiple W or Z chromosomes, or both and sporadic cases of species that have lost the W chromosome [7,26,27]. In some cases, so-called neo-WZ chromosomes have originated by fusion of the ancestral sex chromosomes with a pair of autosomes [11,28,29].

In many lepidopteran species, the W and Z chromosomes are almost indistinguishable in mitotic metaphase, being similar in size and, due to their holokinetic structure (i.e., the absence of a centromere), also resembling autosomes. However, they can usually be differentiated during the pachytene stage of female meiosis, either by the morphology of the WZ bivalent, or with the help of fluorescence in situ hybridization (FISH) [6]. In pachytene, the W and Z chromosomes pair and form a regular bivalent, even if their sequences are highly diverged [30,31]. While the W chromosome is lacking in, or even devoid of, protein-coding genes and is largely composed of heterochromatin, the Z chromosome is gene-rich with an autosome-like appearance [8]. Synteny mapping of Z-linked genes across the major phylogenetic lineages, Tischerioidea plus Ditrysia, suggests a highly conserved gene content of the lepidopteran Z chromosome [32,33,34]. However, due to its hemizygosity in females, the Z chromosome has probably accelerated selective substitution of beneficial mutations (for example, during switching to new host plants, reproductive isolation and speciation, or in developing resistance to insecticides in lepidopteran pests [9,11,13,35]) and also fixation of weakly deleterious mutations by genetic drift. This is called ‘faster-Z evolution’ [36,37]. Attempts to sequence W chromosomes in several species have found mainly mobile elements [38,39,40], whose evolution could also contribute to W chromosomes evolving rapidly and differing greatly even between species in the same family [31,41]. Furthermore, the high density of repetitive sequences can explain the W’s predominantly or entirely heterochromatic state in most species. The sex-determining role of the W chromosome has only recently been proven in a model species, the silkworm *Bombyx mori* [12], while in some other species the W is dispensable [42].

**Figure 1:**
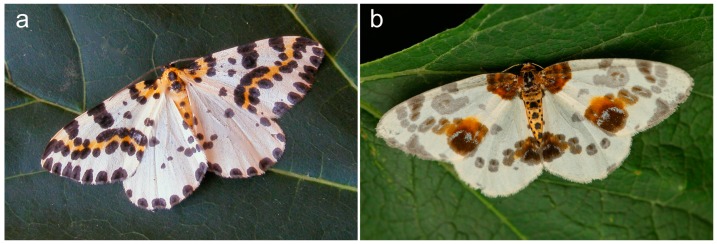
Magpie moth, *Abraxas grossulariata* (**a**) and clouded magpie, *Abraxas sylvata* (**b**). Photographs used with kind permission of Andrej Makara (*Ag*) and Stanislav Krejčík (*As*).

The magpie moth, *A. grossulariata* (Geometridae), with its distinctive speckled wing pattern (Figure 1a), is, as outlined above, the species in which female heterogamety was first discovered. To identify the sex chromosomes and describe their properties, we studied the karyotype of this species along with its congener, the clouded magpie, *A. sylvata* (Figure 1b), using comparative genomic hybridization (CGH) and FISH with W-chromosome painting probes. We also performed a time-calibrated molecular phylogenetic analysis to date the split between the two species.

## 2. Materials and Methods

### 2.1. Insects

The specimens of *A. grossulariata* studied were offspring of two females captured in the Travní Dvůr locality near Hrabětice village in South Moravia, Czech Republic (48°79’ N, 16°43’ E) in June 2011 and June 2015, respectively. After hatching, the larvae were kept on leaves of redcurrant (*Ribes rubrum*) or blackcurrant (*R. nigrum*) at room temperature and natural day length until they reached the third instar and entered diapause. They were then transferred to a protected outdoor area for overwintering. After completion of diapause the larvae were again reared on currant leaves until they reached the penultimate or ultimate larval instar suitable for making male and female meiotic chromosome preparations, respectively. Specimens of *A. sylvata* were offspring of a single female captured near the Kateřinská Cave in the Moravian Karst, Czech Republic (49°36’ N, 16°71’ E) in July 2016. Since in *A. sylvata* the overwintering stage is a pupa, larvae were kept on leaves of the bird cherry (*Prunus padus*) continuously until they reached the penultimate or ultimate larval instar.

### 2.2. Chromosome and Polyploid Nuclei Preparations

Spread chromosome preparations were prepared as described previously [43]. Meiotic chromosomes were obtained from larval gonads, and mitotic preparations were made from larval gonads or wing imaginal discs. Tissues were dissected in a physiological solution designed for *Ephestia* [44]. Wing imaginal discs and male gonads were hypotonized for 10–15 min in 75 mM KCl and then fixed in Carnoy fixative (6:3:1 ethanol, chloroform, acetic acid) for 10–30 min, female gonads were fixed immediately after dissection. Fixed tissues were spread in a drop of 60% acetic acid on the slide at 45 °C using a hot plate. Then preparations were passed through a graded ethanol series (70%, 80%, and 100%, 30 s each) and stored at −20 °C.

Preparations of polyploid interphase nuclei were prepared from Malpighian tubules of male and female larvae as described previously [43]. The tubules were dissected in the same physiological solution as above, fixed in Carnoy fixative for 1 min, and stained in 1.25% lactic acetic orcein for 3–5 min.

### 2.3. Comparative Genomic Hybridization 

Genomic DNA (gDNA) was isolated separately from female and male larvae by standard phenol-chloroform extraction. Genomic DNA probes were labeled using a Nick Translation kit (Abbott Molecular, Des Plaines, IL, USA). The 25 μL nick translation reaction contained 500 ng gDNA; 25 µM dATP, dCTP, and dGTP; 9 µM dTTP; 16 µM labeled nucleotides with either Cy3-dUTP (male gDNA) or fluorescein-12-dUTP (female gDNA) (both Jena Bioscience, Jena, Germany); 1× nick translation buffer and 5 µL of nick translation enzyme mix. The reaction was incubated at 15 °C for 6–7 h.

CGH was performed according to a published protocol [30] with modifications described previously [33]. Briefly, chromosomal preparations were first treated with ribonuclease A (RNase A) (200 ng/μL) (Sigma-Aldrich, St. Louis, MO, USA) in 2× SSC for 1 h at 37 °C and then denatured in 70% formamide in 2× SSC for 3.5 min at 68 °C. The probe mix containing 300 ng of each labeled gDNA probe, 25 μg of sonicated salmon sperm DNA (Sigma-Aldrich) in 10 µL of 50% deionized formamide, 10% dextran sulfate in 2× SSC was denatured for 5 min at 90 °C and prehybridized for 90 min at 37 °C. Hybridization was carried out for three days at 37 °C. Then the slides were washed at 62 °C for 5 min in 0.1× SSC with 1% Triton X-100 and counterstained with 0.5 μg/mL DAPI (4',6-diamidino-2-phenylindole; Sigma-Aldrich) in antifade based on DABCO (1,4-diazabicyclo(2.2.2)-octane; Sigma-Aldrich).

### 2.4. Preparation of W-Chromosome Painting Probes

For laser microdissection of W chromatin bodies, preparations of Malpighian tubules followed a published protocol [39] with slight modifications. The tubules were dissected from the last instar female larvae of both *Abraxas* species in physiological solution, hypotonized for 15 min in 75 mM KCl, fixed in methanol/acetic acid (3:1) for 15 min, transferred into a drop of 60% acetic acid on a glass slide (76 × 24 × 0.17 mm; custom made by Menzel-Gläser, Braunschweig, Germany) coated with 0.0013 mm thick polyethylene naphthalate membrane (Goodfellow, Huntingdon, UK), spread at 40 °C using hot plate and stained with 4% Giemsa (Penta, Prague, Czech Republic). Microdissection of W-bodies was performed using a PALM MicroLaser System (Carl Zeiss MicroImaging, Munich, Germany) as described previously [45].

DNA amplification and probe labeling were performed according to a published method [46]. Briefly, 8-12 sex-chromatin bodies per sample were amplified using GenomePlex Single Cell Whole Genome Amplification Kit (Sigma-Aldrich) and then the reaction was purified by Wizard SV Gel and PCR Clean-Up System (Promega, Madison, WI, USA). The amplified product was labeled using GenomePlex WGA Reamplification Kit (Sigma-Aldrich). The labeling reaction mixture contained 15 ng of amplified DNA, 0.4 mM each dNTP except 0.336 mM dTTP, 40 µM Cy3-dUTP (Jena Bioscience) or Green-dUTP (Abbott Molecular), 1× Amplification mix and 1.7 µL of WGA polymerase in a total volume of 25 µL.

### 2.5. Preparation of Telomeric and rDNA Probes

Insect telomeric probe (TTAGG)*_n_* was synthesized by means of non-template PCR as described previously [47] and labeled with Cy3-dUTP or fluorescein-12-dUTP either by Nick Translation Kit (Abbott Molecular; for details, see above) or using the improved nick translation procedure [48] with some modifications. The modified 20 µL reaction contained 1 µg unlabeled DNA; 50 µM dATP, dCTP, and dGTP; 10 µM dTTP; 20 µM labeled nucleotides; 1× nick translation buffer (50 mM Tris-HCl, pH 7.5; 5 mM MgCl_2_; 0.005% BSA); 10 mM β-mercaptoethanol; 0.005 U DNase I and 20 U DNA polymerase I (both Thermo Fisher, Waltham, MA, USA). The reaction was incubated at 15 °C for 1 h.

An 18S ribosomal DNA (rDNA) probe was generated by PCR from the codling moth (*Cydia pomonella*) gDNA [49] and labeled with biotin-16-dUTP (Roche Diagnostics, Mannheim, Germany) by improved nick translation procedure (for details, see above). The reaction was incubated at 15 °C for 1 h.

### 2.6. Fluorescence In Situ Hybridization with W-Chromosome Painting Probes

FISH was carried out following the protocol for CGH [30] with some modifications. Slides were denatured at 68 °C for 3.5 min in 70% formamide in 2× SSC buffer. For each slide, the probe mixture contained 5 µL of labeled W-chromosome probe and 25 µg of sonicated salmon sperm DNA in a total volume 10 µL of 50% formamide, 10% dextran sulfate in 2× SSC. The probe was denatured at 90 °C for 5 min. Hybridization was carried out for three days at 37 °C. Washes and counterstaining were the same as in the CGH procedure (see above). Probes for cross-species W-painting were hybridized simultaneously, following the same protocol. The quality of each probe was tested individually by hybridization to chromosomes of the original species.

### 2.7. Reprobing

We reprobed the FISH slides after W-painting according to a published protocol [50], in order to verify the localization of major rDNA clusters on the WZ bivalent in both species or to study the chromosomal distribution of telomeric repeats (see below). Briefly, to remove the coverslip and wash away the mounting medium, the slides were immersed in Milli-Q water for 30 min and then washed 2 × 5 min in 2× SSC. Afterwards, to remove the first probe, the slides were incubated for 10 min at 70 °C in 50% formamide, 1% Triton X in 0.1× SSC, then immediately placed in cold 70% ethanol (pre-chilled at −20 °C) for 1 min and dehydrated through 80% and 100% ethanol series for 30 s each and air dried. The slides were immediately used for another round of hybridization.

### 2.8. FISH with Biotin-Labeled 18S rDNA Probe

FISH for localizing major rDNA was performed as described previously [49] with some modifications. Chromosome preparations were first treated with RNase A (200 ng/μL) in 2× SSC for 1 h followed by two washes in 2× SSC for 5 min and 30 min incubation in 5× Denhardt’s solution, all at 37 °C. Chromosomes were denatured in 70% formamide in 2× SSC for 3.5 min at 68 °C. The probe mixture for one slide contained 40 ng of biotin-labeled 18S rDNA probe and 25 μg of sonicated salmon sperm DNA in 10 µL of 50% deionized formamide, 10% dextran sulfate in 2× SSC. Hybridization signals were visualized and amplified by three-step detection, Cy3-conjugated streptavidin (Jackson ImmunoRes. Labs. Inc., West Grove, PA, USA), biotinylated anti-streptavidin (Vector Laboratories, Burlingame, CA, USA) and Cy3-conjugated streptavidin. The preparations were counterstained with 0.5 µg/mL DAPI in DABCO-based antifade.

### 2.9. FISH with Tyramide Signal Amplification (TSA-FISH)

TSA-FISH with the (TTAGG)*_n_* telomeric probe was performed in *A. grossulariata* to examine the presence of interstitial telomeric sequences (ITS) in interstitial heterochromatic blocks. The telomeric probe was prepared by nick translation as described above and purified using Sephadex (Illustra Sephadex G-50 fine DNA grade). TSA-FISH was performed as described previously [51]. Briefly, chromosome preparations after reprobing were treated with 10 mM HCl for 10 min at 37 °C to remove cytoplasm and incubated in 1% hydrogen peroxide for 30 min at room temperature to quench endogenous peroxidase activity. Then the preparations were digested with 100 µg/mL RNase A for 1 h at 37 °C and blocked with 5× Denhardt’s solution for 30 min at 37 °C. Chromosomes were denatured in a probe mix containing 10–30 ng of the labeled telomeric probe, 50% deionized formamide, and 10% dextran sulfate in 2× SSC for 5 min at 70 °C and hybridized overnight. Hybridization signals were enhanced by Antifluorescein-HRP (horseradish peroxidase) conjugate (PerkinElmer, Waltham, MA, USA) diluted 1:1000 and incubated with tyramide solution (TSA Plus Fluorescein system, PerkinElmer) for 5–7 min. The preparations were counterstained and mounted in DABCO-based antifade containing 0.5 µg/mL of DAPI.

### 2.10. Microscopy and Image Processing

Chromosome preparations were observed in a Zeiss Axioplan 2 microscope (Carl Zeiss, Jena, Germany) equipped with a monochrome CCD camera XM10 (Olympus Europa Holding, Hamburg, Germany) and captured separately for each fluorescent dye with cellSens Standard software version 1.9 (Olympus). The images were pseudocolored and merged using Adobe Photoshop CS5 (Adobe Systems, San Jose, CA, USA).

### 2.11. Dating of Split between Abraxas grossulariata and A. sylvata

In recent years, considerable progress has been achieved in both recovering the phylogeny of Lepidoptera and estimating the divergence time of major lineages [52,53]. However, divergence time is currently available only for major clades because a very small fraction of extant taxa was included. This also applies to the diverse family Geometridae, as the extensive dataset [53] included only 11 species of geometrid moths. We used a previous set of timed diversification events [53] as calibration points in a more narrowly focused phylogeny-based analysis to estimate the time of diversification between *A. grossulariata* and *A. sylvata*. 

The DNA sequence data used for calculations were either downloaded from GenBank [54] or were new sequences obtained following previously described protocols [55]. The concatenated data matrix comprised 6473 bp from the mitochondrial *COI* gene and nuclear genes *EF-1a*, *wgl*, *GAPDH*, *RpS5*, *IDH*, *MDH*, and *CAD*. In total, our data matrix includes 22 species from the family Geometridae (including the two *Abraxas* species), four species from the family Uraniidae and two species from the family Sematuridae (Supplementary Table S1). A time-calibrated phylogenetic tree was constructed using Beast 1.8.4 [56]. Details are in Supplementary Text S1.

## 3. Results

### 3.1. Basic Karyotype Characteristics

By examining mitotic metaphases from wing imaginal discs and gonads of male and female larvae, we confirmed that the chromosome number in *Abraxas grossulariata* is 2*n* = 56 (Supplementary Figure S1a), as previously reported [4], while in *A. sylvata* is 2*n* = 58 (Supplementary Figure S1b). In both species, preparations of the larval Malpighian tubules showed a deeply stained sex chromatin body in highly polyploid nuclei of females but not in males (Supplementary Figure S2a–d). The sex chromatin is known to be composed of multiple copies of the W chromosome. Its regular spherical shape and occurrence in females only, together with the same total chromosome number in both sexes, clearly indicate a WZ/ZZ sex chromosome system [6]. The presence of a WZ pair of sex chromosomes in females of both species was confirmed by further research (see below).

**Figure 2:**
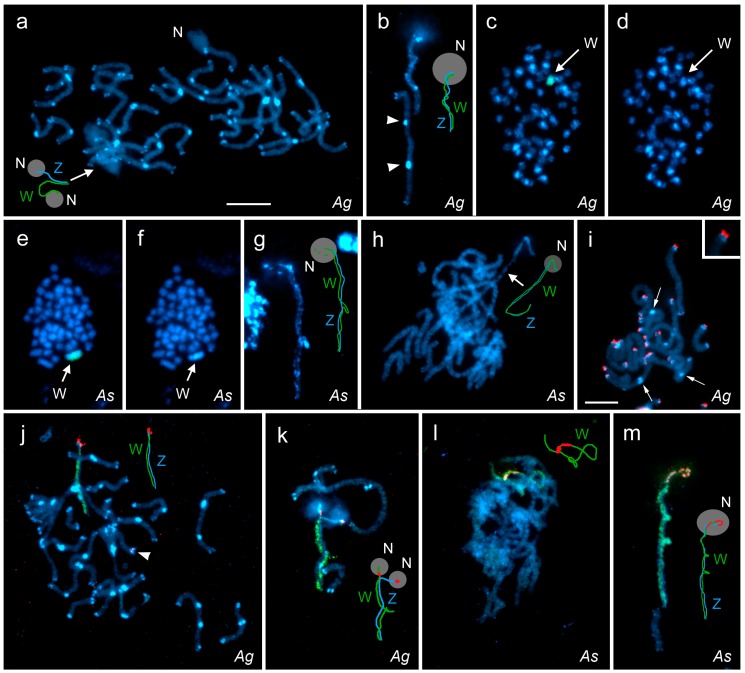
Chromosomes of *Abraxas grossulariata* (*Ag*; **a**–**d**,**i**–**k**) and *A. sylvata* (*As*; **e**–**h**,**l**,**m**) stained with DAPI (blue) and different fluorescence in situ hybridization (FISH) probes. (**a**) Pachytene complement of *Ag*. (**b**) *Ag* WZ bivalent and an autosomal bivalent with heterochromatin blocks on only one homologue (arrowheads). (**c**,**d**) Mitotic metaphase of *Ag* with (**c**) and without (**d**) hybridization signals of the *Ag* W-painting probe (green). (**e**,**f**) Mitotic metaphase of *As* with (**e**) and without (**f**) hybridization signals of the *As* W-painting probe (green). (**g**) *As* WZ bivalent with an interstitial loop on the W chromosome. (**h**) Pachytene complement of *As*. (**i**) Incomplete pachytene nucleus of *Ag* with hybridization signals of the telomeric probe (red) at the ends of bivalents but not in the interstitial heterochromatin blocks (arrows); the inset in the upper right corner shows a detail of the bivalent terminus. (**j**,**k**) Pachytene complement (**j**) and WZ bivalent (**k**) of *Ag* with hybridization signals of the W-painting probe (green) and the 18S ribosomal DNA (rDNA) probe (red); note a pair of small rDNA clusters at the end of an autosomal bivalent (**j**), arrowhead). (**l**,**m**) Pachytene complement (**l**) and WZ bivalent (**m**) of *As* with hybridization signals of the W-painting probe and the 18S rDNA probe. Bar = 10 µm; all figures except (**i**) have the same scale. In the schematic drawings, the green line represents the W chromosome, blue line the Z chromosome, red dots the rDNA in (**j**–**m**), and grey circle the nucleolus (N). The W chromosome in (**a**,**b**,**g**,**h**) was identified by W-painting FISH (hybridization signals not shown). The exact position of the Z chromosome in (**l**) could not be reliably determined, so it is missing in the drawing.

Although the species studied are congeners, their chromosome complements differed greatly. This was evident after simple DAPI staining (cf. Figure 2a,d,f,h), and was particularly well seen in the pachytene stage, where all autosome bivalents in *A. grossulariata*, but not in *A. sylvata*, showed conspicuous terminal blocks of heterochromatin and most of them also had 1–2 large interstitial heterochromatin blocks (Figure 2a). Interestingly, we regularly observed that some interstitial heterochromatic blocks in *A. grossulariata* were present on only one chromosome in the bivalent, suggesting that the individuals were heterozygotes (Figure 2b; Supplementary Figure S3). The W and Z chromosomes each carried a large terminal block of heterochromatin adjacent to the nucleolus, and the W chromosome had several smaller blocks and short segments of heterochromatin (Figure 2a,b), resulting in its DAPI-bright appearance in the highly condensed mitotic chromosomes, although this was not sufficient to identify the W chromosome (Figure 2c,d). 

In contrast, the W chromosome in *A. sylvata* was the only heterochromatic element at mitotic metaphase, and was therefore easily distinguished from other chromosomes, which had no DAPI-bright blocks of heterochromatin (Figure 2e,f). In the WZ bivalent of female pachytene nuclei, we regularly observed numerous small blocks of heterochromatin scattered along the whole W length and two large heterochromatin blocks, one in the middle and one in the subterminal segment associated with the nucleolus (see below). Interestingly, the interstitial block of heterochromatin very often formed one or two loops protruding from the bivalent, where the W chromosome appeared to be locally self-paired (Figure 2g,m). Autosomal heterochromatin blocks occurred only at the ends of some bivalents and were very small and inconspicuous (Figure 2h) compared to those observed in *A. grossulariata* (see Figure 2a). 

The presence of interstitial blocks of heterochromatin in *A. grossulariata* could indicate former chromosome rearrangements, such as inversions and fusions, which would bring otherwise terminal sequences to the interstitial sites. We therefore performed TSA-FISH with a (TTAGG)*_n_* telomeric probe, which could reveal interstitial telomeric sequences (ITS), residues of the former chromosome ends. In pachytene bivalents, typical twin hybridization signals of the probe were observed at the chromosome ends, although their intensity varied greatly from very strong to nearly invisible. However, the probe did not detect any ITS in the massive interstitial heterochromatin blocks (Figure 2i). Moreover, the telomeric hybridization signals often did not co-localize with the terminal blocks of heterochromatin but were next to the blocks at more terminal positions (see the inset in Figure 2i) suggesting that the terminal heterochromatin blocks may in fact be subtelomeric and consist of other repetitive DNA sequences. 

### 3.2. FISH with W-Chromosome Painting Probes and Localization of Major rDNA

The species-specific W-chromosome painting probes highlighted the entire W chromosome in mitotic metaphases of *A. grossulariata* (Figure 2c) and *A. sylvata* (Figure 2e) females. In both species, the W-painting probes also clearly identified the WZ bivalent in female pachytene nuclei (Figure 2a,b,g,h; hybridization signals not shown). FISH with the W-painting probe, followed by FISH with the 18S rDNA probe showed that in both species the WZ bivalent carries the major rDNA clusters, which are located on both the W and Z chromosomes in terminal positions in *A. grossulariata* (Figure 2j,k) and in subterminal positions in *A. sylvata* (Figure 2l,m). In *A. grossulariata*, the rDNA probe revealed another pair of small rDNA clusters located terminally on one autosomal bivalent (Figure 2j). Especially in *A. grossulariata*, the presence of major rDNA clusters at the end of the WZ bivalent apparently impeded proper pairing of this region in pachytene, since the ends of the sex chromosomes were often found unpaired and even formed separate nucleoli (Figure 2k). This also proved that both the W and Z rDNA clusters represent active nucleolar organizer regions (NORs). Also, the remnant of nucleolus observed at the end of the autosomal bivalent suggests the presence of active NORs (Figure 2a). 

### 3.3. Cross-Species W-Chromosome Painting

**Figure 3:**
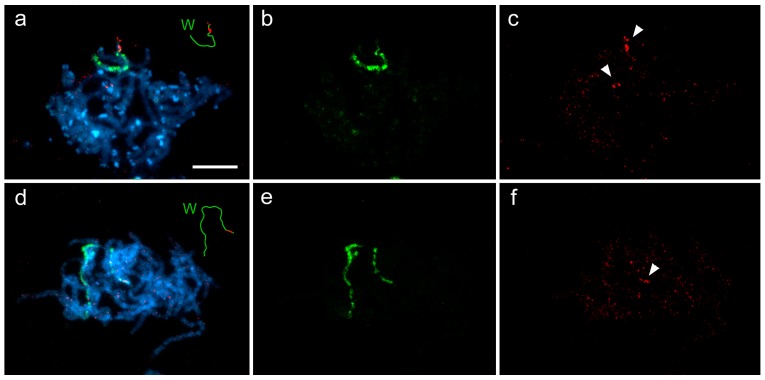
Cross-hybridization of W-chromosome painting probes in pachytene oocytes of *Abraxas grossulariata* (*Ag*) and *A. sylvata* (*As*). Chromosome preparations were counterstained with DAPI (blue). (**a**–**c**) Pachytene complement of *Ag* female: (**a**) merged image of FISH with W-painting probes from *Ag* (green) and *As* (red); (**b**) hybridization signals of the *Ag* W-probe; (**c**) hybridization signals of the *As* W-probe; (**d**–**f**) Pachytene complement of *As* female: (**d**) merged image of FISH with W-painting probes from *As* (green) and *Ag* (red); (**e**) hybridization signals of the *As* W-probe; (**f**) hybridization signals of the *Ag* W-probe. Schematic drawings in (**a**,**d**) show the hybridization pattern of both W- probes on the W chromosome. Arrowheads in (**c**,**f**) indicate hybridization signals of the cross-hybridized W-probe, most likely matching the rDNA clusters. Bar = 10 µm.

To investigate similarities and differences between the W chromosomes of these two congeners, we simultaneously hybridized the differently labeled W-painting probes derived from both species to the female pachytene chromosomes of *A. grossulariata* (Figure 3a–c) and *A. sylvata* (Figure 3d–f). As expected, the W-painting probes from each species labeled the W chromosome of the same species clearly and along the entire length (Figure 3b,e). However, the cross-species hybridizations highlighted only the terminal region of the W chromosome (carrying the NOR), while most of the W chromosome showed weak and scattered hybridization signals comparable to those observed on the autosomes (Figure 3c,f). We conclude that the terminal signals correspond to rDNA because (i) they also labeled the terminal segment of the Z chromosome carrying the NOR, (ii) the coding sequences of rRNA genes are highly conserved in eukaryotes [57] and therefore a high degree of homology is expected between congeners, and (iii) the W-painting probe from *A. sylvata* also showed a pair of autosomal hybridization signals on the *A. grossulariata* chromosomes (Figure 3c), probably matching the autosomal rDNA cluster in this species.

### 3.4. Differentiation of W-Chromosomes by CGH

**Figure 4:**
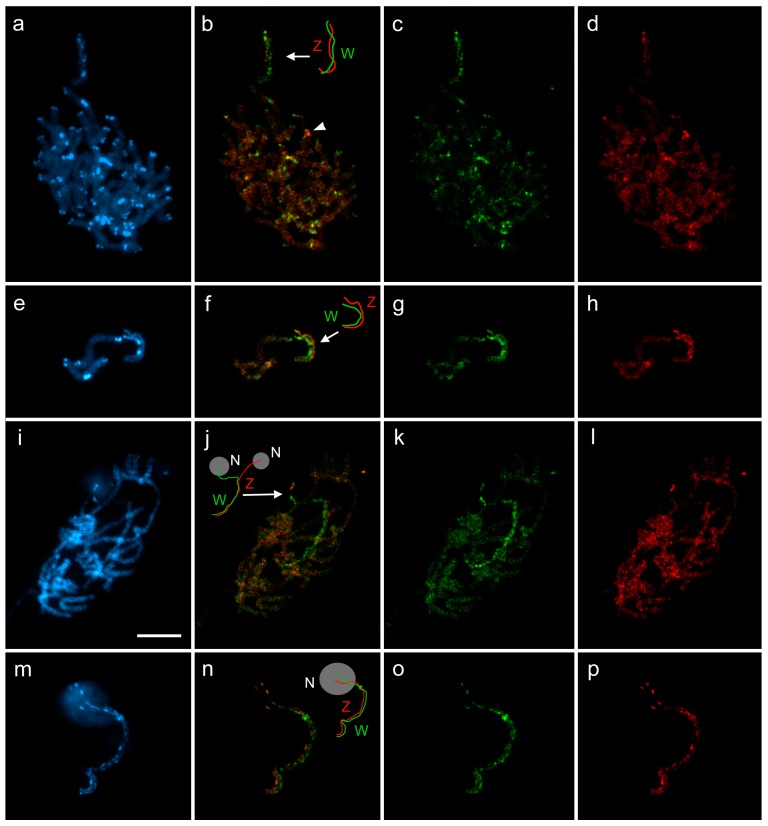
Comparative genomic hybridization on female pachytene chromosomes of *Abraxas grossulariata* (*Ag*); (**a**–**h**) and *A. sylvata* (*As*); (**i**–**p**). Panels (**a**,**e**,**i**,**m**) show DAPI counterstaining (blue), panels (**b**,**f**,**j**,**n**) are merged pictures of hybridization signals of both genomic probes with schematic drawings of the WZ bivalent (W, green line; Z, red line; grey circles are nucleoli), panels (**c**,**g**,**k**,**o**) show hybridization signals of female genomic probes (green), and panels (**d**,**h**,**l**,**p**) show hybridization signals of male genomic probes (red). (**a**–**d**) Pachytene complement of *Ag*. (**e**–**h**) Two autosome bivalents and WZ bivalent of *Ag*. (**i**–**l**) Pachytene complement of *As*. (**m**–**p**) WZ bivalent of *As*. In both species, the female genomic probe strongly highlighted the W chromosome, while the male genomic probe hybridized to all chromosomes equally or slightly more intensively to the Z chromosome as seen in panels (**f**,**h**,**n**,**p**). Note that in *Ag* all heterochromatin blocks were more strongly highlighted by the female probe (**b**), except for a pair of strong hybridization signals of the male probe on one autosomal bivalent (**b**, arrowhead). Bar = 10 µm.

We performed CGH to determine the gross molecular composition of the W chromosome. In both species, male and female genomic probes hybridized to the W chromosome, but stronger labeling with the female probe indicated that the W is enriched with female-specific sequences (Figure 4a–p), except for the NOR-bearing end of the W chromosome, which was equally highlighted by both male and female probes. In addition, the Z chromosome appeared to be more labeled with the male probe (Figure 4f,h,n,p). 

While in *A. sylvata* both male and female genomic probes hybridized evenly with the autosomes, in *A. grossulariata* the probes also highlighted some autosomal heterochromatic blocks. Interestingly, most of these DAPI-bright blocks were slightly more intensively labeled with the female genomic probe, indicating that these regions contain sequences that are enriched on the W chromosome (Figure 4a–d). One autosomal region was strongly labeled with the male genomic probe, probably consisting of repetitive sequences that are abundant on the Z chromosome (Figure 4b, arrowhead). This region was located interstitially on the NOR-bearing autosomal bivalent, but was distant from the terminal rDNA cluster. Surprisingly, unlike the conspicuous DAPI-bright heterochromatin blocks, this region did not differ from the rest of the chromosome after DAPI staining (Figure 4a). 

### 3.5. Dating of Split between Abraxas grossulariata and A. sylvata

The genus *Abraxas* (tribe Abraxini) grouped within the subfamily Ennominae in agreement with the current classification [58]. Its phylogenetic position near the genus *Lomaspilis* (tribe Cassymini) supports the earlier findings [59,60]. Our results suggest that the split between *A. grossulariata* and *A. sylvata* occurred approximately 9.46 million years ago (MYA). The 95% credibility interval for this estimation is 7.18–11.67 million years (Supplementary Figure S4).

## 4. Discussion

In this work, the W and Z sex chromosomes of the iconic magpie moth, *Abraxas grossulariata*, were identified for the first time. They have never been shown before, although the presence of a WZ/ZZ sex chromosome system was indicated by the same chromosome number in males and females (2n = 56), the specific inheritance of the wing pattern typical for sex-linkage in female heterogamety, and the presence of sex chromatin in females [4,61]. We performed a detailed analysis of sex chromosomes in this species and a related species, the clouded magpie, *A. sylvata*, focusing on the molecular evolution of the female-specific W chromosomes.

### 4.1. Sex Chromosome System in Abraxas Moths and Molecular Divergence of Their W-Chromosomes 

Our study confirmed that both *Abraxas* species have the expected WZ/ZZ sex chromosome system. To identify and further characterize their W chromosomes, we applied CGH and FISH with W-chromosome painting probes. CGH enables the identification of W-chromosome regions, consisting of female-specific sequences or regions with sequences shared by both sexes [31]. In some lepidopteran species, female-specific sequences predominate, whereas ubiquitous sequences accumulate in the W chromosome of other species [28,62]. In both *Abraxas* species, CGH revealed that their W chromosomes are mainly composed of female-specific sequences, as found for example in *Tischeria ekebladella* (Tischeriidae) [33]. 

Interestingly, after CGH we observed stronger hybridization signals of the male genomic probe on the Z chromosome of both *Abraxas* species (Figure 4f,h,n,p), which is the theoretically expected result due to the double dose of Z-chromosome-derived sequences in the male genomic probe compared to the female genomic probe. However, this was not observed at CGH experiments performed in other lepidopteran species [31,33,49]. The stronger hybridization signals of the male genomic probe likely result from the abundance of Z-enriched repetitive sequences, which is another unique feature of both *Abraxas* species among Lepidoptera. 

The W chromosome of *A. sylvata* exhibited another interesting feature. It often formed one or two loops in the WZ bivalent, indicating local self-pairing (Figure 2g,m). These W-loops might represent palindromic regions. Palindromes were found in the Y and W chromosomes of some mammals, birds, and *Drosophila*, where they are believed to protect the Y- and W-linked genes from pseudogenization [63]. However, the sequence analysis of several lepidopteran W chromosomes failed to detect protein-coding genes [38,39,40]. Therefore, the role of potential palindromes in the highly heterochromatic W chromosome of Lepidoptera remains elusive.

Although the W chromosomes of both *Abraxas* species consist mainly of female-specific sequences, these sequences differ greatly between the two congeners, as indicated by rare cross-hybridization signals of the W-chromosome painting probes (Figure 3). Our data show that *A. grossulariata* and *A. sylvata* split about 9.5 million years ago (MYA). Given that the lepidopteran W chromosomes seem to be rich in mobile elements [38,39,40], we assume that the molecular divergence of *Abraxas* W chromosomes reflects the independent spreading of female-specific repetitive sequences since the two species split. The dynamics of such molecular divergence has been well demonstrated in the neo-Y chromosomes of different age in *Drosophila* species. During its 15 million years (MY) old history, the neo-Y of *D. pseudoobscura* lost most of its genes and became fully heterochromatic. In *D. miranda*, however, the 1 MY old neo-Y chromosome retains most of its homology with the neo-X chromosome, but ca. 40% of the protein-coding genes are no longer functional due to deleterious mutations, and half of the chromosome consists of mobile elements and is partly heterochromatic. Finally, less than 0.1 MY old neo-Y chromosome in *D. albomicans* shows pseudogenization of only 2% of the protein-coding genes [64]. Although the degeneration seems to be inevitable, its rate changes over time and varies greatly among species being influenced by features such as effective population size, generation length, mode of sex chromosome determination (i.e., XY or WZ), form of dosage compensation, etc. [64,65,66]. For example, well-known and not yet fully explained is a striking difference in the degeneration of W chromosomes between Neognathae and Palaeognathae birds, which have a common origin of more than 130 MYA [67,68,69].

To understand the differences in the rate of degeneration of sex-specific chromosomes, further studies need to be done combining data on molecular differentiation of sex chromosomes with dating splits between related species. However, especially for taxa with WZ systems, such combinations of data are rare. One of the few examples is the fish genus *Triportheus*, in which the W chromosome evolved 15–25 MYA and differentiated in size, morphology, and sequence composition (by accumulation of rDNA, microsatellites, and transposable elements) even between closely related species [70,71,72]. Our results suggest that the W chromosomes of *A. grossulariata* and *A. sylvata* also greatly differentiated from each other during approximately 9.5 MY of independent evolution, except for the region carrying the conservative rDNA. A hallmark of the evolution of sex chromosomes is the restriction of recombination, which is mostly caused by chromosomal inversions that prevent proper meiotic pairing and thus reduce the frequency of crossing-over. Degeneration begins in the inverted regions, leading to loss of genes and the spread of mobile elements [73]. However, Lepidoptera females, like *Drosophila* males, completely lack meiotic recombination [6,64] and, in theory, the degeneration process can begin immediately.

### 4.2. Karyotype Diversification in Abraxas grossulariata and A. sylvata

Our cytogenetic analysis revealed a unique structure of the *A. grossulariata* karyotype (2n = 56) with an extraordinary amount of heterochromatin, forming conspicuous interstitial and terminal blocks in most autosomes. Due to the presence of this heterochromatin, it was virtually impossible to distinguish the W chromosome from other chromosomes in female nuclei using conventional techniques. Such a feature is quite exceptional in lepidopterans because heterochromatin is usually found only in the W chromosome [6,8]. In some species, it is also associated with the NOR [26,28,74], but rarely occurs in other chromosomes [26,27]. These heterochromatin blocks in *A. grossulariata* likely represent sites of high accumulation of repetitive sequences, such as satellite DNA or mobile elements [75]. 

Unlike *A. grossulariata*, *A. sylvata* showed a rather typical lepidopteran karyotype (2n = 58) with heterochromatin-poor autosomes and a heterochromatin-rich W chromosome. Given that in *A. grossulariata* several autosomal blocks of heterochromatin were present in only one homologue (and therefore in the heterozygous state), these blocks must be polymorphic in the population studied (i.e., not fixed), we can infer that the expansion of DNA repeats that created these blocks was a relatively recent event in this species, although more specimens from different populations should be tested, or segregation studied in the progeny of putative heterozygotes. Rapid expansion of repetitive DNA, although common in eukaryotes [76], is known only in a few species of Lepidoptera. For instance, in the butterfly *Leptidea juvernica*, the genome size of populations from eastern Kazakhstan and Ireland, which split ca. 1.6 MYA, differ by ca. 115 Mbp, giving an estimated expansion rate of repetitive DNA in this particular case of ca. 72 Mb per MY [77]. The large heterochromatin content may be one of the reasons why Leonard Doncaster failed to find the heteromorphic pair of sex chromosomes in *A. grossulariata* oocytes despite his thorough cytogenetic investigation [4]. 

While karyotypes of both *Abraxas* species, including their W chromosomes, differ greatly in the heterochromatin content, they share one common feature, i.e., the major rDNA cluster (constituting the active NOR) located at the ends of both W and Z sex chromosomes. In *A. grossulariata*, we found another rDNA cluster at the end of one pair of autosomes. The sex-linkage of rDNA is apparently a rare phenomenon in Lepidoptera, as it was found in only three species [28,78,79]. In both *Abraxas* species, the sex-linkage of rDNA might have arisen by fusion of ancestral sex chromosomes with a pair of NOR-bearing autosomes, although other mechanisms proposed for the rDNA mobility cannot be ruled out [74]. The reduced number of chromosomes compared to the ancestral number of 2n = 62 [32] along with the relatively large size of sex chromosomes in both species support this hypothesis. The origin of such neo-sex chromosomes would probably predated the split of the two *Abraxas* species.

In conclusion, *A. grossulariata* and *A. sylvata* share a WZ/ZZ sex chromosome system, but differ in chromosome number and the number of rDNA clusters. In addition, the autosomes of *A. grossularita* are rich in heterochromatin, whereas the latter species shows heterochromatin only in the W chromosome. This finding suggests that the genomes of both congeners diversified by accumulation of repetitive sequences in *A. grossulariata* but not in *A. sylvata*. Their W chromosomes consist mainly of female-specific sequences that differ greatly between the two species, suggesting a relatively rapid molecular divergence of *Abraxas* W chromosomes during 9.5 MY of independent evolution.

## Supplementary Materials

The following are available online at http://www.mdpi.com/2073-4425/9/6/279/s1. Table S1: Sequences used in the molecular phylogenetic analyses, Table S2: Calibration points used for calculating the age of the split between *Abraxas* spp, Text S1: Phylogenetic analysis, Figure S1: Mitotic chromosomes of *Abraxas* species stained with DAPI, Figure S2: Polyploid nuclei of the Malpighian tubules from *Abraxas* species stained with orcein, Figure S3: Long pachytene bivalents, stained with DAPI, from two specimens of *Abraxas grossulariata*, Figure S4: Timed phylogenetic tree of the Geometroidea.
