# Supplementary material for: Sex Chromosomes of the Iconic Moth Abraxas grossulariata (Lepidoptera, Geometridae) and Its Congener A. sylvata
Source: Genes (Basel). 2018 May 31;9(6):279. doi: 10.3390/genes9060279 (PMC6027526; doi:10.3390/genes9060279)
Supplement: Supplementary file 1 [file genes-09-00279-s001.zip › Zrzava_et_al_genes-09-00279_SupplMat/Supplementary_Figure_S1.pdf]

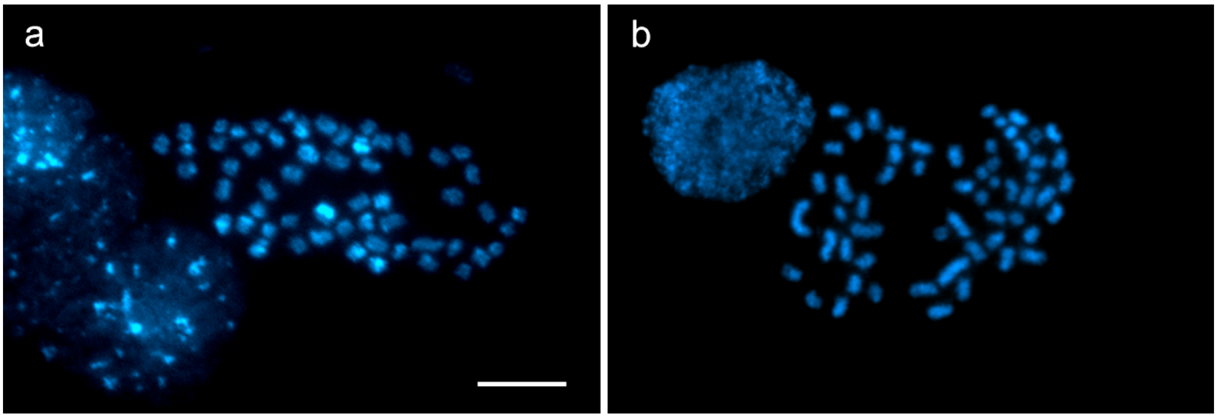

Figure S1. Mitotic chromosomes of *Abraxas* species stained with DAPI. **a** Metaphase of *A. grossulariata* female (2n = 56). **b** Metaphase of *A. sylvata* male (2n = 58). Bar = 10 µm.
