# Supplementary material for: Sex Chromosomes of the Iconic Moth Abraxas grossulariata (Lepidoptera, Geometridae) and Its Congener A. sylvata
Source: Genes (Basel). 2018 May 31;9(6):279. doi: 10.3390/genes9060279 (PMC6027526; doi:10.3390/genes9060279)
Supplement: Supplementary file 1 [file genes-09-00279-s001.zip › Zrzava_et_al_genes-09-00279_SupplMat/Supplementary_Figure_S2.pdf]

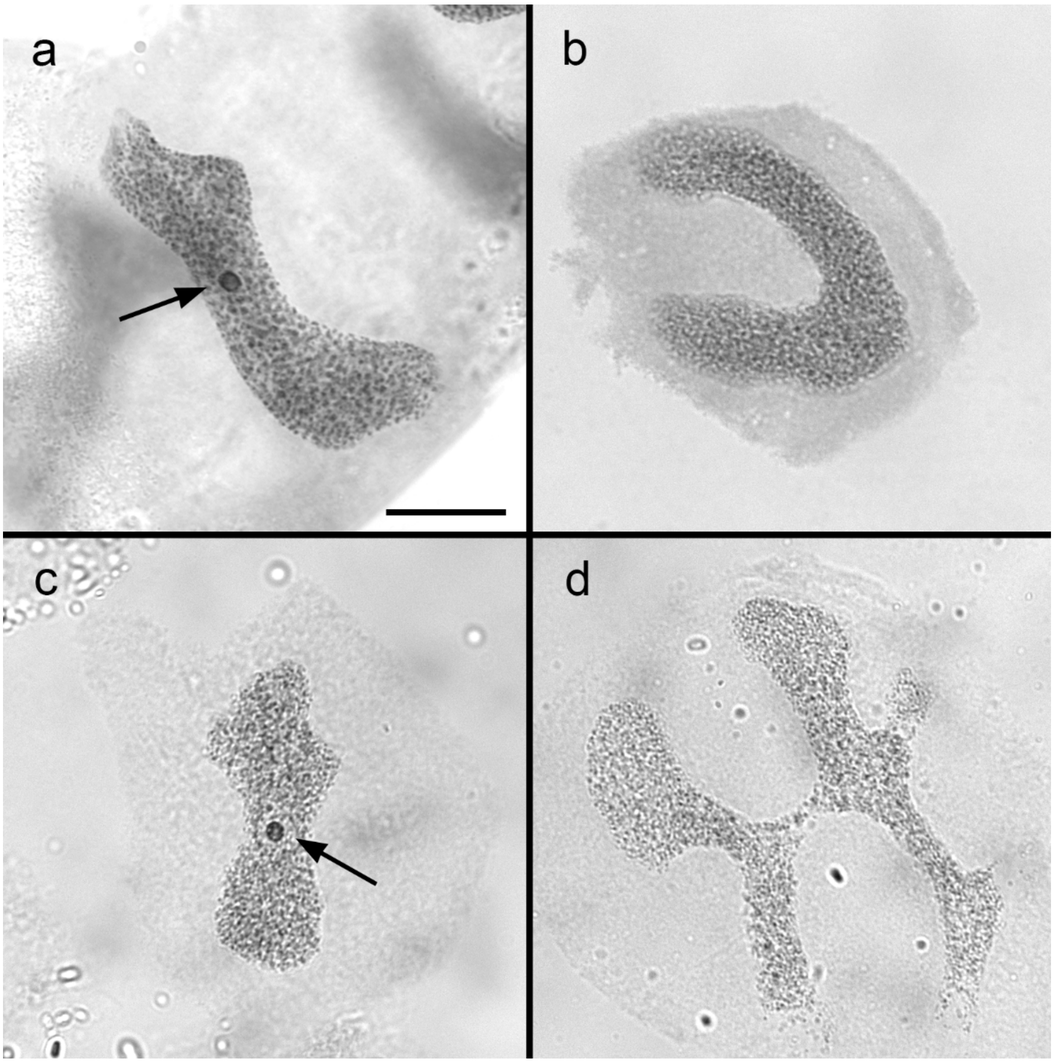

Figure S2. Polyploid nuclei of the Malpighian tubules from *Abraxas* species stained with orcein. *A. grossulariata* female (**a**) and male (**b**), and *A. sylvata* female (**c**) and male (**d**). Note the presence of a conspicuous sex chromatin body (arrows) in female nuclei. Bar = 20 µm.

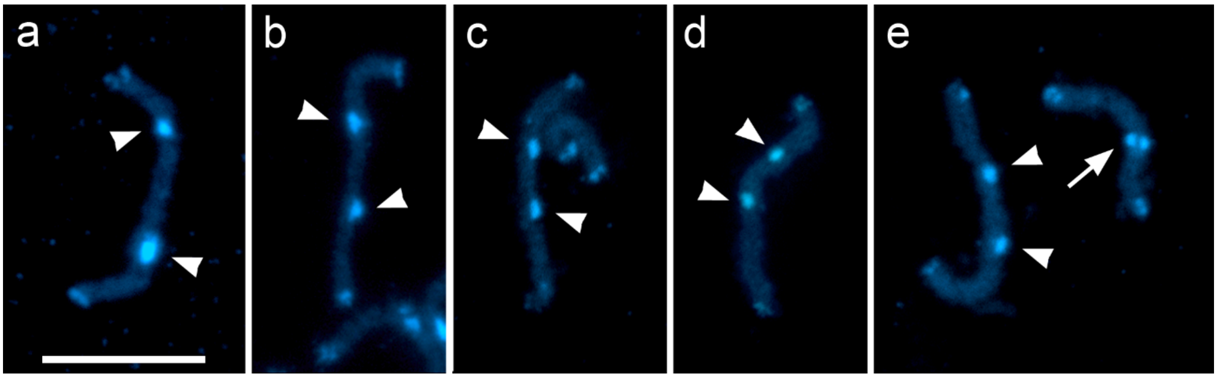

Fig. S3 Long pachytene bivalents, stained with DAPI, from two specimens of *Abraxas grossulariata* (**a**-**e**). Note that each bivalent shows two interstitial blocks of heterochromatin (arrowheads) located on only one homologue of the bivalent, indicating that the specimens were heterozygotes for these heterochromatin blocks. Bivalents in **a** and **e** are from the same nucleus. **e** Comparison of two bivalents: the right bivalent carries a pair of interstitial blocks of heterochromatin (arrow), demonstrating a homozygous state of both homologous chromosomes. Bar = 10 µm

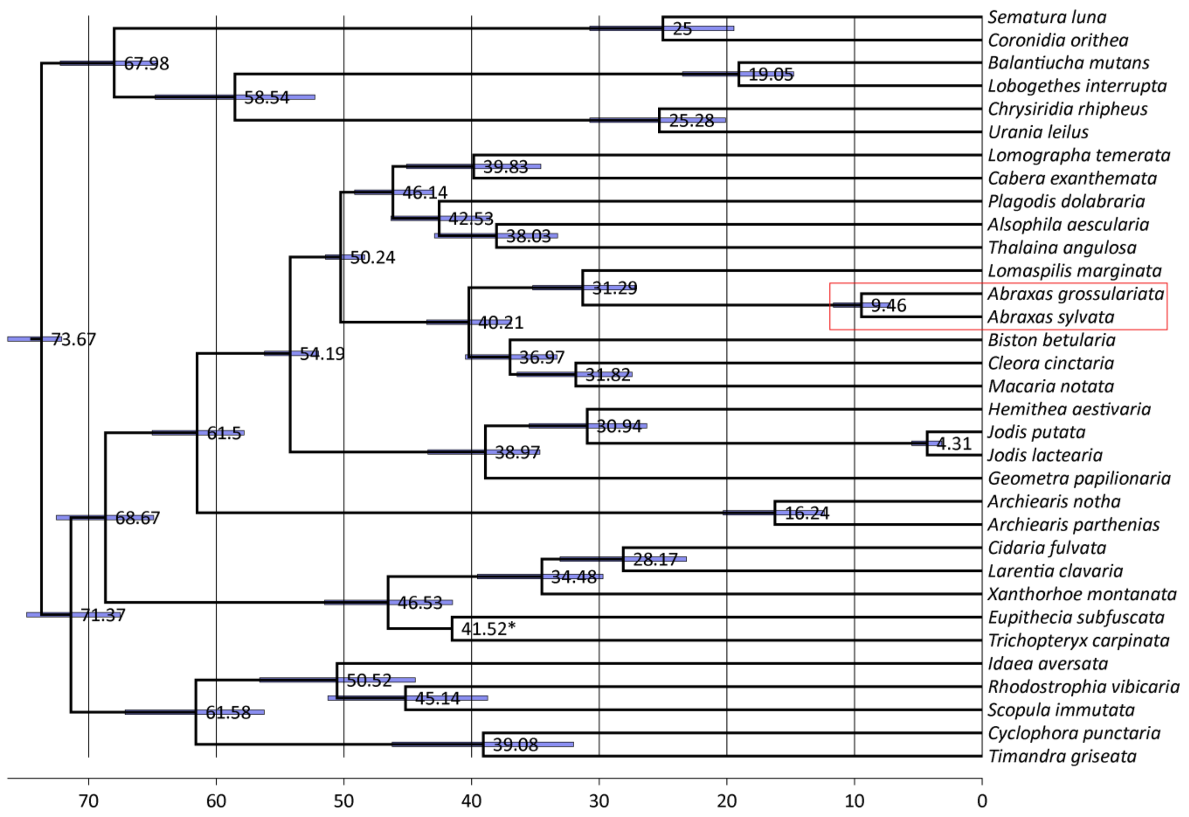

Fig. S4 Timed phylogenetic tree of the Geometroidea. Numbers represent the exact age, and the blue bars indicate 95% confidence interval for each node. Node indicated with an asterisk (*) had very low posterior probability. Target group (*Abraxas* spp.) is indicated by a red quadrangle. Scale: millions of years
