# Supplementary material for: Sex Chromosomes of the Iconic Moth Abraxas grossulariata (Lepidoptera, Geometridae) and Its Congener A. sylvata
Source: Genes (Basel). 2018 May 31;9(6):279. doi: 10.3390/genes9060279 (PMC6027526; doi:10.3390/genes9060279)
Supplement: Supplementary file 1 [file genes-09-00279-s001.zip › Zrzava_et_al_genes-09-00279_SupplMat/Supplementary_Figure_S4.pdf]

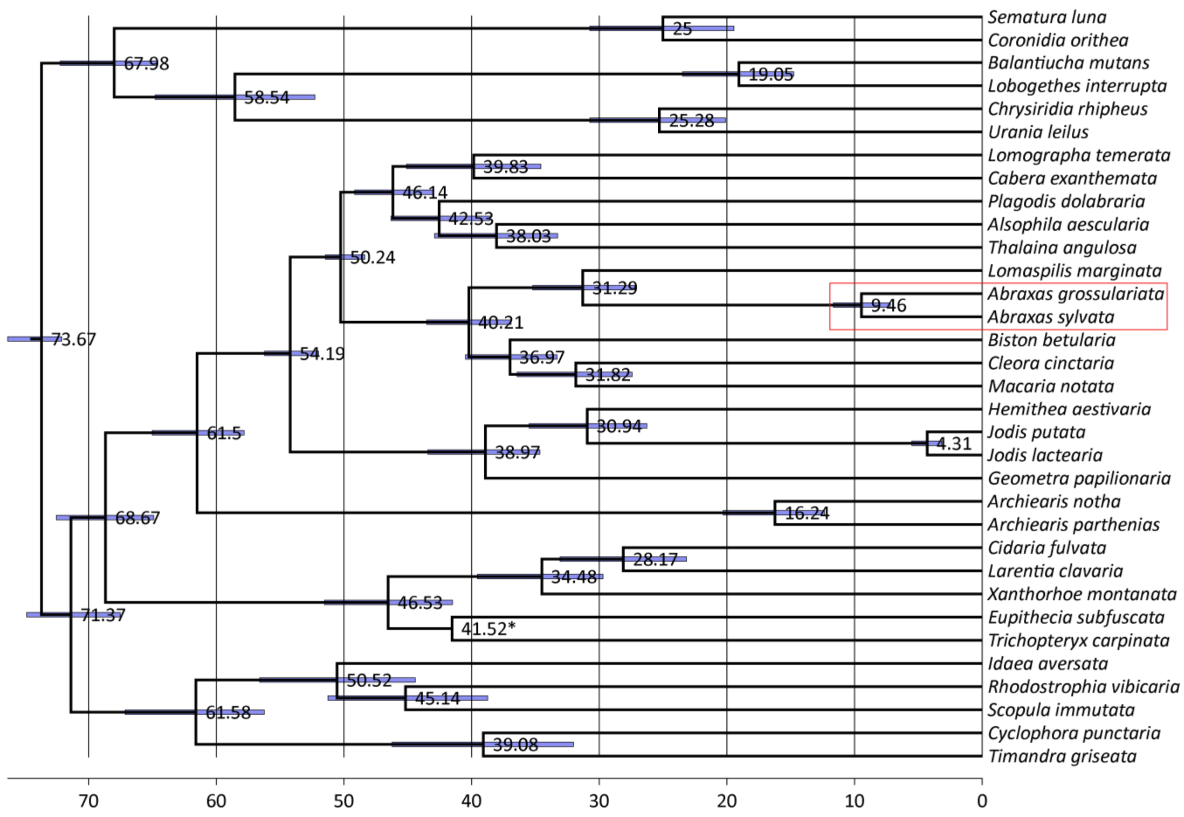

Figure S4. Timed phylogenetic tree of the Geometroidea. Numbers represent the exact age, and the blue bars indicate 95% confidence interval for each node. Node indicated with an asterisk (*) had very low posterior probability. Target group (*Abraxas* spp.) is indicated by a red quadrangle. Scale: millions of years.
