# Supplementary material for: Sex Chromosomes of the Iconic Moth Abraxas grossulariata (Lepidoptera, Geometridae) and Its Congener A. sylvata
Source: Genes (Basel). 2018 May 31;9(6):279. doi: 10.3390/genes9060279 (PMC6027526; doi:10.3390/genes9060279)
Supplement: Supplementary file 1 [file genes-09-00279-s001.zip › Zrzava_et_al_genes-09-00279_SupplMat/Supplementary_Table_S1.pdf]

Table S1. Sequences used in the molecular phylogenetic analyses. Authorships of the sequences downloaded from GenBank are indicated by lowercase letters[a-k] and the relevant publications are listed below. Sequences that were not available for analyses are indicated by ×.

Species	Gene							
	COI	***EF-1α***	***WGL***	***GAPDH***	***RpS5***	***IDH***	***MDH***	***CAD***
**SEMATURIDAE**								
Coronidia orithea	GU828523[c], GU828327[c]	GU828869[c], GU829171[c]	×	GU829715[c]	GU830559[c]	GU829914[c]	GU830247[c]	GU828035[c]
Sematura luna	GU828862[c], GU929824[c]	GU829165[c], GU829427[c]	×	GU829912[c]	GU830809[c]	GU830240[c]	GU830554[c]	GU828322[c]
**URANIIDAE**								
Balantiucha mutans	GU828806[c], GU929776[c]	GU829111[c], GU829388[c]	GU829665[c]	GU829881[c]	×	GU830187[c]	GU830505[c]	GU828278[c]
Lobogethes interrupta	GU828807[c], GU929777[c]	GU829112[c], GU829389[c]	GU829666[c]	GU829882[c]	×	GU830188[c]	GU830506[c]	GU828279[c]
Urania leilus	DQ018927[j]	DQ018898[j]	DQ018868[j]	×	EU141430[i]	EU141589[i]	EU141653[i]	EU141351[i]
Chrysiridia rhipheus	GU828839[c], GU929803[c]	GU829140[c], GU829410[c]	GU829690[c]	GU829900[c]	GU830788[c]	GU830219[c]	GU830530[c]	JF785274[g]
**GEOMETRIDAE**								
**Archiearinae**								
Archiearis notha	KX343539[f]	KX343683[f]	KX343624[f]	×	KX343275[f]	KX343349[f]	×	×
Archiearis parthenias	KX343538[f]	DQ018899[j]	KX343623[f]	EU141485[i]	EU141381[i]	EU1415392[i]	EU141604[i]	JF785273[g]
**Geometrinae**								
Geometra papilionaria	KX343540[f]	GU580809[k]	KX343625[f]	GU580821[k]	GU830656[c]	GU830046[c]	KX343746[f]	KX343819[f]
Hemithea aestivaria	KX788614[a]	KX788718[a]	JF785123[g]	JF785454[g]	JF785007[g]	JF785531[g]	JF784898[g]	JF785256[g]
Jodis lactearia	MG767763[b]	MG768161[b]	MG767317[b]	MG767567[b]	MG767394[b]	MG768382[b]	MG768003[b]	MG768546[b]
Jodis putata	KX343541[f]	KX343684[f]	GU593342[k]	KX343408[f]	JF784987[g]	GU580853[k]	KX343747[f]	KX343820[f]
**Ennominae**								
Abraxas grossulariata	HQ340174[e]	HQ340207[e]	HQ340234[e]	HQ340216[e]	×	JF785523[g]	×	JF785240[g]
Abraxas sylvata	MH423813*	GU580800[k]	GU593336[k]	GU580830[k]	GU580668[k]	MH423823*	MH423826*	MH423829*

Alsophila aescularia	GU580755[k]	GU580794[k]	GU593332[k]	×	×	GU580856[k]	GU830326[c]	×
Biston betularia	EF206681[h]	EF206673[h]	GU829589[c]	GU580832[k]	GU580670[k]	GU580867[k]	GU830417[c]	JF785230[g]
Cabera exanthemata	KX788605[a]	KX788710[a]	×	JF785452[g]	JF785001[g]	JF785509[g]	JF784892[g]	JF785216[g]
Cleora cinctaria	GU580763[k]	GU580801[k]	GU593337[k]	GU580831[k]	GU580669[k]	GU580866[k]	JF784867[g]	JF785215[g]
Lomaspilis marginata	GU580777[k]	GU580813[k]	×	JF785434[g]	GU580685[k]	GU580877[k]	JF784865[g]	JF785212[g]
Lomographa temerata	MH423814*	HQ340210[e]	HQ340237[e]	MH423818*	HQ340228[e]	HQ340220[e]	×	MH423830*
Macaria notata	MH423815*	MH423816*	MH423817*	MH423819*	MH423821*	MH423824*	MH423827*	MH423831*
Plagodis dolabraria	KX788622[a]	KX788726[a]	KX788764[a]	KX788747[a]	KX788788[a]	KX788643[a]	KX788809[a]	KX788598[a]
Thalaina angulosa	GU828797[c], GU929767[c]	GU829103[c], GU829382[c]	GU829656[c]	GU829875[c]	×	GU830178[c]	GU830498[c]	GU828271[c]
**Larentiinae**								
Cidaria fulvata	KX343553[f]	KX343692[f]	KX343632[f]	KX343421[f]	KX343286[f]	KX343360[f]	KX343759[f]	KX343832[f]
Eupithecia subfuscata	KX343576[f]	KX343709[f]	KX343647[f]	KX343437[f]	KX343306[f]	KX343374[f]	KX343773[f]	KX343851[f]
Larentia clavaria	KX343583[f]	KX343715[f]	KX343653[f]	KX343443[f]	KX343313[f]	GU830070[c]	GU830387[c]	JF785226[g]
Trichopteryx carpinata	EU443349[d]	EU443287[d]	EU443308[d]	KX343461[f]	KX343335[f]	KX343395[f]	KX343806[f]	JF785229[g]
Xanthorhoe montanata	KX343615[f]	KX343738[f]	KX343675[f]	KX343468[f]	KX343342[f]	KX343402[f]	KX343812[f]	KX343887[f]
**Sterrhinae**								
Cyclophora punctaria	EU443361[d]	EU443298[d]	EU443318[d]	×	KX788769[a]	KX788629[a]	KX788791[a]	KX788584[a]
Idaea aversata	EU443357[d]	EU443294[d]	EU443315[d]	KX343410[f]	KX343277[f]	KX343351[f]	KX343749[f]	KX343822[f]
Rhodostrophia vibicaria	EU443354[d]	EU443292[d]	EU443313[d]	×	KX788771[a]	KX788630[a]	KX788792[a]	KX788585[a]
Scopula immutata	KX788627[a]	KX788731[a]	KX788767[a]	MH423820*	MH423822*	MH423825*	MH423828*	MH423832*
Timandra griseata	EU443362[d]	EU443299[d]	EU443319[d]	KX788733[a]	KX788772[a]	KX788631[a]	JF784893[g]	JF785252[g]

* This study

[a] Holm, S.; Davis, R.B.; Javoiš, J.; Õunap, E.; Kaasik, A.; Molleman, F.; Tammaru, T. A comparative perspective on longevity: the effect of body size dominates over ecology in moths. *J. Evol. Biol.* **2016**, 29, 2422–2435. doi: 10.1111/jeb.12966

[b] Holm, S.; Javoiš, J.; Õunap, E.; Davis, R.; Kaasik, A.; Molleman, F.; Tasane, T.; Tammaru, T. Reproductive behaviour indicates specificity in resource use: phylogenetic examples from temperate and tropical insects. *Oikos* **2018**. First published: 27 February 2018. doi: 10.1111/oik.04959

[c] Mutanen, M.; Wahlberg, N.; Kaila, L. Comprehensive gene and taxon coverage elucidates radiation patterns in moths and butterflies. *Proc. Biol. Sci.* **2010**, 277, 2839–2848. doi: 10.1098/rspb.2010.0392

[d] Õunap, E.; Viidalepp, J.; Saarma, U. Systematic position of Lythriini revised: transferred from Larentiinae to Sterrhinae (Lepidoptera, Geometridae). *Zool. Scr.* **2008**, 37, 405–413.

[e] Õunap, E.; Javoiš, J.; Viidalepp, J.; Tammaru, T. Phylogenetic relationships of selected European Ennominae (Lepidoptera: Geometridae). *Eur. J. Entomol.* **2011**, 108, 267–273. doi: 10.14411/eje.2011.036

[f] Õunap, E.; Viidalepp, J.; Truuverk, A. Phylogeny of the subfamily Larentiinae (Lepidoptera: Geometridae): integrating molecular data and traditional classifications. *Syst. Entomol.* **2016**, 41, 824–843. doi.org/10.1111/syen.12195

[g] Sihvonen, P.; Mutanen, M.; Kaila, L.; Brehm, G.; Hausmann, A.; Staude, H.S. Comprehensive molecular sampling yields a robust phylogeny for geometrid moths (Lepidoptera: Geometridae). *PLoS One* **2011**, 6, e20356. doi: 10.1371/journal.pone.0020356

[h] Viidalepp, J.; Tammaru, T.; Snäll, N.; Ruohomäki, K.; Wahlberg, N. *Cleorodes* Warren, 1894 does not belong in the tribe Boarmiini (Lepidoptera: Geometridae). *Eur. J. Entomol.* **2007**, 104, 303–309. doi: 10.14411/eje.2007.046

[i] Wahlberg, N.; Wheat, C.W. Genomic outposts serve the phylogenomic pioneers: designing novel nuclear markers for genomic DNA extractions of Lepidoptera. *Syst. Biol.* **2008**, 57, 231–242. doi: 10.1080/10635150802033006

[j] Wahlberg, N.; Braby, M.F.; Brower, A.V.Z.; de Jong, R.; Lee, M.M.; Nylin, S.; Pierce, N.E.; Sperling, F.A.H.; Vila, R.; Warren, A.D.; Zakharov, E. Synergistic effects of combining morphological and molecular data in resolving the phylogeny of butterflies and skippers. *Proc. Biol. Sci.* **2005**, 272, 1577–1586. doi: 10.1098/rspb.2005.3124

[k] Wahlberg, N.; Snäll, N.; Viidalepp, J.; Ruohomäki, K.; Tammaru, T. The evolution of female flightlessness among Ennominae of the Holarctic forest zone (Lepidoptera, Geometridae). *Mol. Phylogenet. Evol.* **2010**, 55, 929–938. doi: 10.1016/j.ympev.2010.01.025
