# Supplementary material for: Sex Chromosomes of the Iconic Moth Abraxas grossulariata (Lepidoptera, Geometridae) and Its Congener A. sylvata
Source: Genes (Basel). 2018 May 31;9(6):279. doi: 10.3390/genes9060279 (PMC6027526; doi:10.3390/genes9060279)
Supplement: Supplementary file 1 [file genes-09-00279-s001.zip › Zrzava_et_al_genes-09-00279_SupplMat/Supplementary_Table_S2.pdf]

**Table S2.** Calibration points used for calculating the age of the split between *Abraxas* spp. Numbers indicate the start value and the upper and lower boundaries of the prior following uniform distribution for each calibration point.

| Taxon set | First run | | Final run | |
|---|---|---|---|---|
| | Start value | Boundaries | Start value | Boundaries |
| Geometroidea | 82.7 | 72.1-93.0 | 82.7 | 72.1-93.0 |
| Uraniidae+Sematuridae | 76.2 | 64.6-87.9 | 76.2 | 64.6-87.9 |
| Geometridae | 71.5 | 60.9-82.6 | 71.5 | 60.9-82.6 |
| Archiearinae+Geometrinae+Ennominae | 60.5 | 49.0-71.5 | 60.6 | 49.0-71.5 |
| Geometrinae+Ennominae | 46.1 | 35.8-56.2 | 46.1 | 35.8-56.2 |
| Geometrinae | Using tree prior | Using tree prior | Using tree prior | Using tree prior |
| Ennominae | Using tree prior | Using tree prior | 40.9 | 30.6-51.4 |
| *Alsophila+Thalaina* | Taxon set not defined | Taxon set not defined | 32.9 | 21.4-43.6 |
| *Abraxas grossulariata+Abraxas sylvata* | Taxon set not defined | Taxon set not defined | Using tree prior | Using tree prior |
