# Supplementary material for: Sex Chromosomes of the Iconic Moth Abraxas grossulariata (Lepidoptera, Geometridae) and Its Congener A. sylvata
Source: Genes (Basel). 2018 May 31;9(6):279. doi: 10.3390/genes9060279 (PMC6027526; doi:10.3390/genes9060279)
Supplement: Supplementary file 1 [file genes-09-00279-s001.zip › Zrzava_et_al_genes-09-00279_SupplMat/Supplementary_Text_S1.pdf]

Text S1. Phylogenetic analysis.

For phylogenetic reconstruction, we first determined the best partitioning scheme from three competing scenarios. For the first scenario, each gene was defined as a separate partition; for the second scenario, each codon position (1st, 2nd and 3rd) of each gene was defined as a separate partition; for the third scenario, the data were divided between 50 subsets (bins) according to their evolutionary rates using Tiger v 1.02 (Cummins and McInerney 2011). To choose between these scenarios, a maximum likelihood (ML) tree following GTR+I+G model was first constructed using Mega6 (Tamura et al. 2013). This tree was used as user_tree_topology in PartitionFinder 1.1.1 (Lanfear et al. 2012) to find the best partitioning scheme for all three competing scenarios. Bayesian Information Criterion (BIC) values of the best scheme for each scenario were compared and the bin-based treatment was selected for further calculations.

Beast 1.8.4 (Drummond et al. 2012) was used to calculate the time of the split between *Abraxas* spp. According to the suggestion by PartitionFinder, nucleotide data were divided into four partitions, each comprising one or more bins. HKY+G, GTR, GTR+G and GTR+I+G models were each implemented on one partition according to suggestions by PartitionFinder. Relaxed molecular clock allowing branch lengths to vary according to uncorrelated lognormal distribution (Drummond et al. 2006) was used for each partition. The taxon sampling within Geometridae was very limited in Wahlberg et al. (2013). Therefore, we first needed to reveal the phylogenetic position of *Abraxas* in order to make the most of calibration data from Wahlberg et al. (2013). For the preliminary analysis, seven calibration points were defined. For five of those, a uniform prior was used within 95% confidence interval from Wahlberg et al. (2013) to define their age, whereas for two calibration points no prior assumptions were made (for details, see Supplementary Table S2). The tree prior was set to the Birth-Death process (Gernhard 2008). All other priors were let to defaults. Four independent analyses were performed, each with Bayesian MCMC running for 10 million generations and sampling every 1000th generation. The results were inspected in Tracer 1.5 (available from http://tree.bio.ed.ac.uk/software/tracer/) and 1 million generations were discarded from all runs. Thereafter, results of all analyses were combined together using LogCombiner v1.6.2 (a supplementary software to Beast). A summary tree was constructed using TreeAnnotator v1.6.2 (also a supplementary software to Beast) and visualized in FigTree v1.4.0 (http://tree.bio.ed.ac.uk/software/figtree/). Based on this initial tree, the phylogenetic position of *Abraxas* was taken into account for calculating the age of the split between *A. grossulariata* and *A. sylvata*. Two more calibration points were defined and the number of timed calibration points was also increased by two (for details, see Supplementary Table S2) for the final analysis. All other details of the final analysis were identical to the initial run described above. The final tree is presented in Supplementary Figure S4.

References

Cummins, C.A.; McInerney, J.O. A method for inferring the rate of evolution of homologous characters that can potentiallyimprove phylogenetic inference, resolve deep divergence and correct systematic biases. *Syst. Biol.* **2011**, 60, 833–844. doi: 10.1093/sysbio/syr064

Drummond, A.J.; Ho, S.Y.W.; Phillips, M.J.; Rambaut, A. Relaxed phylogenetics and dating with confidence. *PLoS Biol.* **2006**, 4, 699–710. doi: 10.1371/journal.pbio.0040088

Drummond, A.J.; Suchard, M.A.; Xie, D.; Rambaut, A. Bayesian phylogenetics with BEAUti and the BEAST 1.7. *Mol. Biol. Evol.* **2012**, 29, 1969–1973. doi: 10.1093/molbev/mss075

Gernhard, T. The conditioned reconstructed process. *J. Theor. Biol.* **2008**, 253, 769–778. doi: 10.1016/j.jtbi.2008.04.005

Lanfear, R.; Calcott, B.; Ho, S.Y.W.; Guindon, S. PartitionFinder: combined selection of partitioning schemes and substitution models for phylogenetic analyses. *Mol. Biol. Evol.* **2012**, 29, 1695–1701. doi: 10.1093/molbev/mss020

Tamura, K.; Stecher, G.; Peterson, D.; Filipski, A.; Kumar, S. MEGA6: Molecular Evolutionary Genetics Analysis Version 6.0. *Mol. Biol. Evol.* **2013**, 30, 2725–2729. doi: 10.1093/molbev/mst197

Wahlberg, N.; Wheat, C.W.; Peña, C. Timing and patterns in the taxonomic diversification of Lepidoptera (butterflies and moths). *PLoS One* **2013**, 8, e80875.
doi: 10.1371/journal.pone.0080875
